# Knowledge and Practice Related to COVID-19 and Mental Health among Adults in Sub-Saharan Africa

**DOI:** 10.4269/ajtmh.21-0219

**Published:** 2021-06-23

**Authors:** Firehiwot Workneh, Dongqing Wang, Ourohiré Millogo, Alemayehu Worku, Angela Chukwu, Bruno Lankoande, Nega Assefa, Elena C. Hemler, Michelle L. Korte, Abdramane Soura, Ayoade Oduola, Ali Sie, Wafaie W. Fawzi, Yemane Berhane

**Affiliations:** 1 Department of Epidemiology and Biostatistics, Addis Continental Institute of Public Health, Addis Ababa, Ethiopia;; 2 Department of Global Health and Population, Harvard T.H. Chan School of Public Health, Harvard University, Boston, Massachusetts;; 3 Nouna Health Research Center, Nouna, Burkina Faso;; 4 Department of Preventive Medicine, School of Public Health, College of Health Sciences, Addis Ababa University, Addis Ababa, Ethiopia;; 5 Department of Statistics, University of Ibadan, Ibadan, Nigeria;; 6 Institut Supérieur des Sciences de la Population, Université de Ouagadougou, Ouagadougou, Burkina Faso;; 7 College of Health and Medical Sciences, Haramaya University, Harar, Ethiopia;; 8 Institut Supérieur des Sciences de la Population, University of Ouagadougou, Ouagadougou, Burkina Faso;; 9 University of Ibadan Research Foundation, University of Ibadan, Ibadan, Nigeria

## Abstract

Coronavirus disease 2019 (COVID-19) is a public health emergency affecting the lives of millions of people globally. Different measures and extraordinary steps are being taken to contain the transmission of the virus. The levels of knowledge and implementation of preventive practices related to COVID-19 in sub-Saharan African countries are unclear. Additionally, there is a lack of evidence regarding the impacts of the pandemic on mental health. This study aimed to describe knowledge and practices related to COVID-19 and to assess mental health status among adults in three sub-Saharan African countries: Burkina Faso, Ethiopia, and Nigeria. A total of 1,797 adults were included in the survey, and data were collected using computer-assisted telephone interviews. The proportions of adults who identified more than 80% of COVID-19 symptoms, transmission methods, and prevention mechanisms were 69.9%, 79.2%, and 90.7%, respectively. The practice of preventive measures was relatively lower for avoiding social gatherings and disinfecting contaminated surfaces. Better education, urban residence, and believing the pandemic is real were factors associated with good knowledge on COVID-19 symptoms, transmission methods, and preventive actions. Additionally, being male was associated with good knowledge on symptoms and transmission methods, whereas being in an older age group was associated with knowledge of transmission methods. Mild, moderate, and severe psychological distress was reported by 20.6%, 5.9%, and 1.1% of the participants, respectively. Although this study found high levels of knowledge regarding COVID-19, interventions are needed to increase the uptake of recommended preventive practices among adults in sub-Saharan Africa.

## INTRODUCTION

In December 2019, the coronavirus disease (COVID-19) caused by the SARS-CoV-2 virus emerged in the Chinese city of Wuhan.^1^ Due to its exponential growth and high case fatality rate, the WHO declared COVID-19 as a public health emergency of international concern in January and as a pandemic in March 2020.^2,3^ Public health interventions, including active cases detection, isolation of cases, contact tracing and quarantine, social distancing, and community quarantine, were advocated as control measures for COVID-19.^4,5^ Accordingly, governments enforced travel and movement restrictions, prohibited gatherings, instituted generalized or partial lockdown, and promoted measures to improve hygiene and prevent further transmission of the virus.

The pandemic was projected to be catastrophic in sub-Saharan Africa (SSA) due to fragile health systems in many countries, high burden of other diseases, limited economic resources, and social and cultural specificities.^6,7^ The risk of severe consequences in these contexts urged health authorities to rapidly apply interventions to induce behavioral changes to reduce transmission risk at an early stage in the pandemic.^8,9^ Public health measures include social distancing, use of personal protective equipment, and handwashing.^10^ However, the public health measures adopted to control the spread of the pandemic (especially measures such as lockdowns) may have affected the mental health status of the general population in many settings either by exacerbating existing or triggering new mental health conditions.^11–13^

Communities’ compliance with public health interventions to control the outbreak relies on individual perceptions of risk and knowledge.^14^ To support public decision-makers to be efficient in allocating resources, defining communication messages, and designing robust interventions, evidence is needed on COVID-19 knowledge and practices among the general population.

The pandemic has the potential to have a significant impact on population mental health in SSA. Previous studies have reported an increase in mental health problems among the general population following epidemics or pandemics.^15,16^ Evidence suggests a rise in the number of new cases and exacerbation of previous mental health problems globally following the COVID-19 pandemic.^13^ Mental health is also an important consideration for COVID-19 response because it affects people’s behaviors and emotions, which in turn affects compliance to the recommended public health safety measures.^16,17^ It also affects people’s adaptation and ability to cope with changes in regulations. Pandemic-related mental health problems may be caused by mitigation measures such as lockdowns, quarantine, social isolations, and movement restrictions.^15,16,18,19^

Elevated levels of stress and anxiety so far are the largest public mental health impacts of the pandemic. Other negative mental health conditions, including post-traumatic stress disorders, depression, suicide, and harmful alcohol and substance use, are predicted to increase with the expansion of the socioeconomic impacts of the pandemic.^20–22^ Disease experience, stigma, and discrimination are related to short-term outcomes, whereas socio-economic impacts such as losses of job/income and disruptions to daily routines are correlated with long-term mental health conditions.^13,22,23^ With the rapid increase of COVID-19 cases, there is a strong concern that the mental health impacts of COVID-19 will be serious in SSA due to the region’s weak health care systems and low uptake of mental health services.^13,24^ Given this potential burden in the SSA region, evidence is needed to determine the scale of the problem and strengthen mental health and psychosocial support as part of the COVID-19 response.

This study aimed to collect data among adults in three SSA countries to assess knowledge and practices related to COVID-19 and to measure levels of psychological distress. Understanding knowledge and practices surrounding COVID-19 and its impact on other health domains among the general population is necessary to inform preventive strategies and innovative interventions to mitigate the direct and indirect health consequences of the pandemic.

## MATERIALS AND METHODS

### Study sites and study population.

This study was conducted in three countries in SSA: Burkina Faso, Ethiopia, and Nigeria. Burkina Faso has a population of 20.9 million and is among the countries with the lowest literacy rates in Africa (41.2%) in 2020.^25^ The capital city, Ouagadougou, which has a population of 1,475,223 million, was selected as the urban site for this study, and the Ouagadougou Health and Demographic Surveillance Site (HDSS) was used as the sampling frame. Nouna, a town in the Kossi province, was selected as the rural site. The Nouna HDSS covers a population of 89,718 and was used as the sampling frame for the Nouna site.

Ethiopia is the second most populous nation in Africa and has an adult literacy rate of 51.77%. A greater proportion of the population lives in rural areas (79%); the remaining 21% are urban dwellers.^25,26^ The urban site for this study, Addis Ababa, is the capital and is a densely populated city with more than 3.35 million people. The environmental conditions in the city include overcrowding in squalid housing and neighborhoods, poor sanitation, and air pollution.^27^ The rural site for this survey was Kersa, Ethiopia, which is a small rural district in the Oromia region. The Kersa HDSS currently covers a population of approximately 131,000 individuals and was used as the sampling frame for this site.

The third country included in the survey is Nigeria, the most populous country in Africa with an adult literacy rate of 60.2%.^25^ Ibadan is the capital of the Oyo State in Nigeria and is composed of both urban and rural areas. Households in rural areas of Ibadan included in the Nigeria Living Standards Survey made up the sampling frame for the rural site in Nigeria. Lagos, the largest city in Nigeria and one of the largest cities in SSA, was selected as the urban site in Nigeria for this study. Telephone numbers from households located in urban areas of Lagos were obtained from telephone service providers to make up the sampling frame for the household survey.

Among all African countries, Ethiopia and Nigeria were ranked as fourth and seventh, respectively, according to numbers of COVID-19 cases as of November 2020, contributing to 12% of the total cases in the African continent. Data were collected in Burkina Faso in August–September 2020. In September 2020, Burkina Faso had 2,032 confirmed COVID-19 cases and 58 deaths due to COVID-19, with a daily average of 10 or less in August and 20 or more in September. In Ethiopia, data collection for the two sites spanned September through November 2020. In Ethiopia, the daily new cases peaked in August and September, with a daily average as high as 1,510 cases. Cases were relatively lower in October compared with the previous 2 months, and the total confirmed cases were 96,169, with 1,469 deaths by October 31, 2020. In Nigeria, data were collected between October to November 2020, and the number of COVID-19 cases was 522,673, with 7,816 deaths by the end of November.

Sites were selected based on existing data collection infrastructure, previous experience working in study sites, and research team capacity and willingness to participate. Detailed survey methods and further information regarding the sites included have been published elsewhere.^28^

#### Study design and sampling.

This study was a cross-sectional mobile phone survey conducted among adults in three SSA countries. Approximately 600 adults residing in urban and rural sites were included in the survey in each country (300 per site). In each household, one adult aged 20 years or older was interviewed. In each survey site, available population-based platforms were used to construct sampling frames to select households for participation in the survey. Existing Health and Demographic Surveillance Systems were used as sampling frames in Kersa, Ethiopia and in Nouna and Ouagadougou, Burkina Faso. In Nigeria, the National Living Standards Survey 2018–2019 and lists from telephone service providers were used to obtain a sampling frame. In Addis Ababa, a new household survey was established. We randomly selected 2,500 households from each urban and rural site to allow us to reach the required sample size of 300 adults. Participants with any working phone were included in this survey. During the actual data collection, both mobile and landline phones were used, but the majority of participants were contacted using their mobile phones. The interviews were conducted mainly during the day, but phone calls were also made at night to accommodate participants’ schedules.

#### Data collection.

In compliance with COVID-19 protocols in each country, this survey used Computer-Assisted Telephone Interviewing to collect survey data. Data were collected by trained research staff using the site-specific local languages. Data were collected from August to November 2020 in all six sites.

A standardized questionnaire developed in consultation with subject matter experts at participating institutions across the three countries was used for data collection. For this study, we assessed 1) sociodemographic information; 2) knowledge, practices, and perceptions of COVID-19; and 3) mental health and COVID-19. The full questionnaire used has been published elsewhere.^28^ For the psychological distress measurement, the ultra-brief Patient Health Questionnaire (PHQ)-4 tool was used, which is a validated tool that combines two questions from the PHQ-9 and two questions from the Generalized Anxiety Disorder-7. The four questions in the PHQ-4 tool capture the core symptoms of depression and anxiety. The specific questions have been validated for brief screening of self-reported depression and anxiety.^29–32^

The questionnaire, which was developed initially in English, was translated into local languages by the research team at each site. Practicability, validity, and interpretability of answers for the respective questions was confirmed by performing a pretest among adults in the six survey sites. Based on the pre-test, slight modifications were made to the tool to refine it for each specific context. During the phone interview, data collectors entered participant data into a mobile tablet-based data collection system, Open Data Kit. Data were uploaded to a secure server in each country after collection.

#### Ethical approval.

This study obtained ethical approval from the institutional review boards of Harvard T.H. Chan School of Public Health and the ethical authorities in each country (National Ethics Committee and Nouna Health Research Center Ethical Committee in Burkina Faso, Institutional Ethical Review Board of Addis Continental Institute of Public Health in Ethiopia, and National Health Research Ethics Committee and University of Ibadan Research Ethics Committee in Nigeria). Verbal informed consent was obtained from each study participant before conducting the interviews.

#### Statistical analysis.

Stata V14 statistical software was used for data cleaning and analysis. Basic descriptive statistics, such as counts and percentages for categorical variables, means and SDs for normally distributed continuous variables, and medians and interquartile ranges for continuous variables with skewed distributions, were calculated by survey site. Knowledge related to COVID-19 was assessed in three domains: symptoms, transmission, and prevention. For each of the three domains, a score was created by adding the correct responses of the variables included for the respective domains. A higher score indicates better knowledge in that domain.

The total possible scores for the three knowledge domains of symptoms, transmission, and prevention were 10, 5, and 7, respectively. The cutoffs to be considered having reasonably good knowledge were 8, 4, and 6, respectively, representing an accuracy of 80% or more in correctly identifying the particular domain, which is consistent with Bloom’s cutoff point.^33–36^ A total COVID-19 knowledge score was created by adding up the scores of the three domains. The scores ranged from 0 to 22, with a cutoff score of 18 representing an accuracy of 80%.

Logistic regression models were used to assess bivariate and multivariable associations between dependent and independent variables. Variables that showed significant association in the bivariate analysis and other potential confounders that showed association with COVID-19 knowledge in the literature were used to construct the multivariable model.^37–40^ The statistical significance level was set at *P* < 0.05.

The PHQ-4 was used to measure depression and anxiety among adults. The two anxiety questions—”Feeling nervous, anxious or on edge” and “Not being able to stop or control worrying”—were asked using a four-scale response of “Not at all,” “Several days,” “More than half the days,” and “Nearly every day” over the past 2 weeks. Participants were allowed to refuse or choose the option of “don’t know,” which was not used in this analysis. The depression questions—“Feeling down, depressed or hopeless,” and “Little interest or pleasure in doing things”—were also asked using the same answer options as the anxiety questions. The four possible responses were coded 0–3 for each of the four questions. Anxiety and depression subscales were created using the specific questions, and each had a range of 0–6. For each of the subscales, a score of 3 or greater was considered as having high levels of anxiety and depression. A psychological distress scale was then created by adding the four questions, which added up to a maximum score of 12 and a minimum score of 0. The total score for depression/anxiety was categorized as none (total score: 0–2), mild (total score: 3–5), moderate (total score: 6–8), and severe (total score: 9–12).^29,41,42^

## RESULTS

### Characteristics of the study participants.

A total of 1,797 adults were interviewed across the three countries, with approximately 300 participants per site. The majority of the respondents (63.4%) were male. The mean age was 42.4 years, ranging from 20 to 90 years. Compared with the other sites, Burkina Faso had the highest proportion of participants in the older age group (38.7% and 37.7% were > 50 years in Nouna and Ouagadougou, respectively), and this proportion was lowest in Kersa (7.4%) (Table 1). Close to one-third (28.1%) of participants were not literate; 23.3% had completed tertiary education or higher. Most of the adults interviewed (75.8%) were household heads, and the proportion engaged in farming was higher for the rural sites, mainly for Nouna and Kersa.

**Table 1:** Characteristics of the survey participants in Burkina Faso, Ethiopia, and Nigeria, 2020

		Burkina Faso		Ethiopia		Nigeria	
Characteristics	Total	Nouna	Ouagadougou	Addis Ababa	Kersa	Ibadan	Lagos
Number of participants	1,797	297	300	288	297	304	311
Sex (*N* = 1,797), *N* (%)							
Female	658 (36.6)	11.8 (35)	32.0 (96)	64.6 (186)	22.2 (66)	51.3 (156)	38.3 (119)
Male	1,139 (63.4)	88.2 (262)	68.0 (204)	35.4 (102)	77.8 (231)	48.7 (148)	61.7 (192)
Age* (*N* = 1,710), *N* (%)							
20–29	230 (13.5)	14 (4.7)	7 (2.3)	71 (24.6)	7 (11.1)	51 (18.7)	54 (21.1)
30–39	496 (29)	62 (20.9)	50 (16.7)	105 (36.5)	149 (50.2)	66 (24.3)	64 (25)
40–49	550 (32.2)	106 (35.7)	130 (43.3)	53 (18.40	93 (31.3)	83 (30.5)	85 (33.2)
≥ 50	434 (25.4)	115 (38.7)	113 (37.7)	59 (20.5)	22 (7.4)	72 (26.5)	53 (20.7)
Mean (±SD)	42.4 (±12.3)	48.4 (±13.1)	47.3 (±9.9)	38.8 (±12.6)	36.7 (±7.6)	41.4 (±12.2)	40.8 (±12.9)
Educational status† (*N* = 1,780), *N* (%)							
None, religious school, literacy class	500 (28.1)	183 (61.6)	174 (58.0)	29 (10.1)	106 (35.7)	6 (2.0)	2 (0.7)
Some primary school education	278 (15.6)	46 (15.5)	39 (13.0)	73 (25.3)	115 (38.7)	5(1.7)	0 (0.0)
Completed primary school	194 (10.9)	36 (12.1)	31 (10.3)	27 (9.4)	40 (13.5)	45 (15.0)	15 (4.9)
Some secondary/high school	185 (10.4)	23 (7.7)	50 (16.7)	45 (15.6)	23 (7.7)	39 (13.0)	5 (1.6)
Completed secondary/high school	189 (10.6)	3 (1.0)	1 (0.3)	43 (14.9)	11 (3.7)	82 (27.2)	49 (16.1)
Tertiary education (vocational training, college, university) or higher	434 (24.4)	6 (2.0)	5 (1.7)	70 (24.3)	2 (0.7)	121 (40.2)	230 (75.7)
Occupational status‡ (*N* = 1,797), *N* (%)							
Unemployed	89 (4.9)	1 (0.3)	26 (8.7)	56 (19.4)	0 (0.0)	3 (0.9)	3 (0.9)
Student	95 (5.3)	14 (4.7)	1 (0.3)	11 (3.8)	10 (3.4)	33 (10.9)	26 (8.4)
Farmer	529 (29.4)	226 (76.1)	24 (8)	0 (0.0)	265 (89.2)	9 (2.9)	5 (1.6)
Wage employee	275 (15.3)	13 (4.4)	47 (15.7)	29 (10.1)	4 (1.4)	72 (23.7)	110 (35.4)
Self-employed	531 (29.6)	21 (7.1)	133 (44.3)	77 (26.7)	3 (1.0)	168 (55.3)	129 (41.5)
Stay-at-home parent	110 (6.1)	5 (1.7)	31 (10.3)	44 (15.3)	26 (8.7)	0 (0.0)	4 (1.3)
Casual, off farm income	55 (3.6)	2 (0.7)	28 (9.3)	12 (4.0)	0 (0.0)	10 (3.3)	3 (0.9)
Other	106 (5.9)	7 (2.4)	20 (6.7)	9 (3.1)	6 (2.0)	26 (8.6)	38 (12.2)
Household head (*N* = 1,797), *N* (%)		297	300	288	294	296	232
No	475 (25.4)	13.1 (39)	13.3 (40)	21.2 (61)	14.8 (44)	49.3 (150)	39.5 (123)
Yes	1,340 (74.6)	86.9 (258)	86.7 (260)	78.8 (227)	85.2 (253)	50.7 (154)	60.5 (188)
Household size§ (*N* = 1,776), *N* (%)		297	300	288	294	296	232
≤ 5	823 (46.3)	46 (15.5)	76 (25.3)	230 (79.9)	75 (25.3)	186 (62.4)	210 (70.9)
6–10	786 (44.3)	153 (51.5)	186 (62)	58 (20.1)	208 (70.0)	101 (33.9)	80 (27.0)
≥ 11	167 (9.4)	98 (33)	38 (12.7)	0 (0.0)	14 (4.7)	11 (3.7)	6 (2.0)
Mean (±SD)	6.5 [±3.5]	9.9 (±4.9)	7.3 (±2.9)	4.2 (±1.7)	6.9 (±2.1)	5.3 (±2.5)	4.6 (±1.9)

* Information was missing for 32 participants in Ibadan and 55 participants in Lagos.

† Counts and percentages do not add up to the total because the selection of multiple responses was allowed.

‡ Information was missing for 3 participants in Ibadan and 7 participants in Lagos.

§ Information was missing for 6 participants in Ibadan and 15 participants in Lagos.

#### Knowledge of COVID-19 symptoms, transmission methods, and preventive actions and associated factors.

The majority of respondents had good knowledge of the three COVID-19 knowledge domains, knowledge on symptoms, transmission methods, and preventive actions. A large portion of the respondents were able to correctly identify the common COVID-19 symptoms (headache, fever, cough, muscle weakness, sore throat, and shortness of breath), which was similar across the six sites (Table 2). According to the score and classification for this domain, 69.9% had a good knowledge of COVID-19 symptoms. The median score was 8 (Q1: 7; Q3: 8) overall and was higher in Burkina Faso, which had a median score of 9 (Table 3). Being male, residing in an urban area, and believing the pandemic is real were associated with good knowledge of COVID-19 symptoms (Table 4).

**Table 2:** Description of knowledge on symptoms, transmission, and preventive actions on COVID-19 in Burkina Faso, Ethiopia, and Nigeria, 2020

Knowledge domains	Total	Burkina Faso		Ethiopia		Nigeria	
		Nouna	Ouagadougou	Addis Ababa	Kersa	Ibadan	Lagos
Symptoms of COVID-19, *N* (%)							
Headache	1,462 (82.0)	257 (87.4)	263 (87.9)	240 (83.3)	270 (91.2)	220 (74.1)	212 (68.8)
Fever	1,577 (88.3)	264 (89.2)	280 (93.3)	266 (92.4)	281 (94.9)	230 (77.2)	256 (83.1)
Cough	1,598 (90.0)	265 (89.2)	286 (95.3)	265 (92.0)	288 (97.3)	234 (79.6)	260 (86.7)
Muscle weakness	1,453 (81.9)	236 (79.5)	251 (83.7)	229 (79.5)	262 (88.5)	224 (75.7)	251 (84.2)
Sore throat	1,468 (82.8)	237 (79.8)	263 (87.7)	265 (92.0)	232 (78.6)	220 (75.1)	251 (83.4)
Runny nose	1,367 (77.1)	254 (85.8)	267 (89.3)	197 (68.4)	259 (88.4)	196 (62.2)	194 (64.2)
Muscle and joint aches	1,283 (72.3)	222 (74.8)	222 (74.0)	188 (65.3)	244 (83.9)	196 (66.7)	211 (69.2)
Shortness of breath	1,546 (86.6)	253 (85.5)	258 (86.0)	257 (89.2)	258 (87.8)	243 (81.5)	277 (89.6)
Loss of smell	1,181 (66.2)	201 (67.7)	195 (65.0)	162 (56.3)	200 (68.0)	183 (61.6)	240 (77.7)
Rash	757 (42.5)	127 (42.8)	190 (63.3)	113 (39.2)	49 (16.6)	127 (43.0)	151 (49.4)
Transmission methods, *N* (%)							
Through respiratory droplets in the air from infected persons	1,671 (93.9)	262 (88.2)	291 (79.3)	284 (98.6)	285 (96.3)	265 (91.1)	284 (92.5)
Through objects and surfaces contaminated with the virus	1,698 (94.7)	259 (87.2)	291 (97.0)	283 (98.3)	294 (99.0)	280 (92.7)	291 (93.8)
Through physical contact with an infected person	1,683 (93.9)	249 (83.8)	293 (97.7)	279 (96.9)	291 (98.3)	283 (94.0)	288 (92.9)
Through mosquito bites	816 (45.7)	83 (27.9)	138 (46.0)	91 (31.6)	77 (26.1)	189 (63.4)	238 (77.0)
Through cellular mobile networks	1,433 (80.0)	210 (70.9)	246 (82.0)	230 (79.7)	263 (88.6)	240 (79.7)	244 (78.9)
Preventive actions, *N* (%)							
Stay at home when not working	1,552 (86.8)	223 (75.3)	265 (88.3)	283 (98.3)	269 (91.2)	243 (81.3)	269 (86.8)
Put distance (at least 2 meters)	1,720 (96.1)	274 (92.3)	287 (96.0)	282 (97.9)	293 (98.7)	281 (93.7)	303 (98.4)
Wash hands often with soap and running water	1,748 (97.6)	284 (95.9)	293 (97.7)	286 (99.3)	291 (98.0)	288 (96.0)	306 (98.7)
Use hand sanitizer	1,739 (97.4)	285 (95.9)	295 (98.3)	285 (98.9)	283 (96.6)	287 (96.0)	304 (98.4)
Cover cough and sneezes	1,725 (96.5)	282 (94.9)	294 (98.0)	285 (98.9)	286 (96.6)	280 (93.7)	298 (96.8)
Wear a mask	1,734 (97.3)	286 (96.3)	298 (99.3)	283 (98.3)	281 (96.2)	287 (95.6)	299 (97.7)
Drink alcohol	1,218 (68.4)	158 (52.2)	231 (77.0)	221 (76.7)	233 (79.5)	186 (62.6)	189 (61.7)
Information sources on COVID-19,^*^ *N* (%)							
Radio	1,426 (79.4)	267 (89.9)	269 (89.7)	109 (37.9)	278 (93.6)	259 (85.2)	244 (78.5)
Television	1,322 (73.6)	247 (83.2)	218 (72.7)	264 (91.7)	93 (31.3)	231 (76)	269 (86.5)
Government messages	767 (42.7)	69 (23.2)	33 (11.0)	157 (54.5)	146 (49.2)	179 (58.9)	183 (58.8)
Friends/family	753 (41.9)	155 (52.2)	181 (60.3)	81 (28.1)	62 (20.9)	111 (36.5)	163 (52.4)
Social media (e.g., Facebook, WhatsApp)	528 (29.4)	31 (10.4)	55 (18.3)	35 (12.2)	10 (3.4)	162 (53.3)	235 (75.6)
Newspapers	343 (19.1)	40 (13.5)	14 (4.7)	7 (2.4)	0 (0.0)	107 (35.2)	175 (56.3)
Search on the internet	288 (16.0)	3 (1.0)	9 (3.0)	48 (16.7)	3 (1.0)	75 (24.7)	150 (48.2)

* Counts and percentages do not add up to the total because the selection of multiple responses was allowed.

**Table 3:** Knowledge scores on the three domains across the survey sites in Burkina Faso, Ethiopia, and Nigeria, 2020

		Burkina Faso		Ethiopia		Nigeria	
Knowledge scores	Total	Nouna	Ouagadougou	Addis Ababa	Kersa	Ibadan	Lagos
Knowledge on symptoms (0–10), %							
Poor (< 8)	30.1	27.5	23.1	33.3	26.5	39.4	31.7
Good (≥ 8)	69.9	72.5	76.9	66.7	73.5	60.6	68.3
Median (25th p, 75th p)^*^	8 (7, 9)	9 (7, 9)	9 (8, 10)	8 (7, 9)	8 (7, 9)	8 (6, 9)	8 (7, 9)
Knowledge on transmission (0–5), %							
Poor (< 4)	20.8	39.2	16.4	19.8	12.6	21.4	15.7
Good (≥ 4)	79.2	60.8	83.6	80.2	87.4	78.6	84.3
Median (25th p, 75th p)	4 (4, 5)	4 (3, 4)	4 (4, 5)	4 (4, 5)	4 (4, 4)	5 (4, 5)	5 (4, 5)
Knowledge on prevention (0–7), %							
Poor (< 6)	9.3	16.9	8.0	2.8	5.7	14.3	9.3
Good (≥ 6)	90.7	83.1	92.0	97.2	94.3	85.7	90.7
Median (25th p, 75th p)	7 (6, 7)	6 (6, 7)	7 (6, 7)	7 (6, 7)	7 (6, 7)	6 (6, 7)	6 (6, 7)
Total knowledge on COVID-19 (0–22), %							
Poor (< 18)	25.4	32.6	17.6	21.9	21.6	36.9	22.9
Good (≥ 18)	74.6	67.4	82.4	78.1	78.4	63.1	77.1
Median (25th p, 75th p)	19 (17, 20)	18 (17, 20)	19 (18, 21)	19 (18, 20)	19 (18, 20)	19 (16, 20)	19 (18, 20)

* 25th p = 25th percentile; 75th p = 75th percentile.

**Table 4:** Results of logistic regression analysis of factors associated with knowledge on symptoms, transmission methods, and preventive actions of COVID-19 in Burkina Faso, Ethiopia, and Nigeria, 2020

	Knowledge on symptoms		Knowledge on transmission methods		Knowledge on preventive actions	
Variables	COR (95% CI)	AOR (95% CI)	COR (95% CI)	AOR (95% CI)	COR (95% CI)	AOR (95% CI)
Sex						
Female	1.0		1.0		1.0	
Male	1.75 (1.49–2.07)^*^	1.59 (1.26–2.01)^*^	1.21 (0.96–1.54)	1.34 (1.03–1.75)^*^	0.87 (0.62–1.22)	1.15 (0.78–1.67)
Age, years						
20–29	1.0		1.0		1.0	
30–39	1.25 (0.89–1.77)	1.15 (0.79–1.65)	0.71 (0.47–1.09)	0.80 (0.52–1.25)	0.74 (0.39–1.38)	0.85 (0.44–1.65)
40–49	1.20 (0.86–1.69)	0.98 (0.68–1.41)	0.72 (0.47–1.08)	0.76 (0.49–1.19)	0.69 (0.37–1.29)	0.74 (0.39–1.43)
≥ 50	1.22 (1.55–1.74)	1.04 (0.71–1.52)	0.54 (0.36–0.83)^*^	0.63 (0.40–0.98)^*^	0.48 (0.26–0.89)^*^	0.60 (0.31–1.16)
Educational status						
None, religious school, literacy class	1.0		1.0		1.0	
Some primary school education	1.25 (0.89–1.75)	1.29 (0.91–1.83)	1.45 (1.02–2.07)^*^	1.37 (0.95–1.99)^*^	1.89 (1.12–3.21)^*^	1.76 (1.02–3.05)^*^
Completed primary school	0.81 (0.57–1.16)	0.86 (0.57–1.25)	1.17 (0.79–1.72)	1.17 (0.95–1.77)	1.05 (0.64–1.73)	1.04 (0.61–1.78)
Some secondary/high school	0.97 (0.67–1.42)	1.05 (0.71–1.58)	1.67 (1.09–2.57)^*^	1.67 (1.05–2.63)^*^	2.33 (1.20–4.52)^*^	2.41 (1.15–5.09)^*^
Completed secondary/high school	0.75 (0.53–1.09)	1.11 (0.72–1.72)	1.24 (0.83–1.85)	1.21 (0.76–1.94)	1.33 (0.78–2.29)	1.32 (0.69–2.54)^*^
Tertiary education (vocational training, college, university) or higher	0.96 (0.72–1.28)	1.02 (0.72–1.44)	2.41 (1.72–3.40)^*^	2.08 (1.39–3.11)^*^	2.61 (1.59–4.29)^*^	2.01 (1.13–3.60)^*^
Place of residence						
Rural	1.0		1.0		1.0	
Urban	1.08 (0.88–1.33)	1.27 (1.01–1.61)^*^	3.09 (2.05–4.74)^*^	1.50 (1.16–1.96)^*^	2.13 (1.52–2.99)^*^	2.21 (1.49–3.24)^*^
Believe COVID-19 pandemic is real						
Yes	1.0		1.0		1.0	
No	2.68 (1.75–1.38)^*^	2.69 (1.73–4.22)^*^	3.09 (2.02–4.74)^*^	3.16 (2.01–4.97)^*^	3.34 (2.01–5.55)^*^	3.56 (2.07–6.12)^*^
Family size						
≤ 5	1.0		1.0		1.0	
6–10	1.19 (0.96–1.48)	1.12 (0.87–1.45)	0.96 (0.75–1.23)	1.21 (0.91–1.61)	0.93 (0.66–1.32)	1.34 (0.88–2.02)
≥ 11	1.28 (0.87–1.86)	1.22 (0.79–1.87)	0.56 (0.38–0.81)^*^	0.82 (0.53–1.27)	0.50 (0.31–0.82)^*^	0.92 (0.51–1.65)

AOR = adjusted odds ratio; COR = crude odds ratio.

* *P* value < 0.05

More than 90% of participants identified the three main COVID-19 transmission mechanisms, which were transmission through respiratory droplets in the air from infected persons, objects and surfaces contaminated with the virus, and physical contact with an infected person. Over half believed in transmission misconceptions, including the transmission of COVID-19 through mosquito bites, which was especially high in the rural sites of Nouna and Kersa (Table 2). The median score for knowledge of COVID-19 transmission was 4 (Q1: 4; Q3: 5) and was similar across the six survey sites (Table 3). Male sex, older age group (≥ 50), educational status (having some primary school, some secondary or tertiary education compared with no education), urban residence, and believing the pandemic is real were positively associated with knowledge of COVID-19 transmission mechanisms (Table 4).

**Figure 1.**
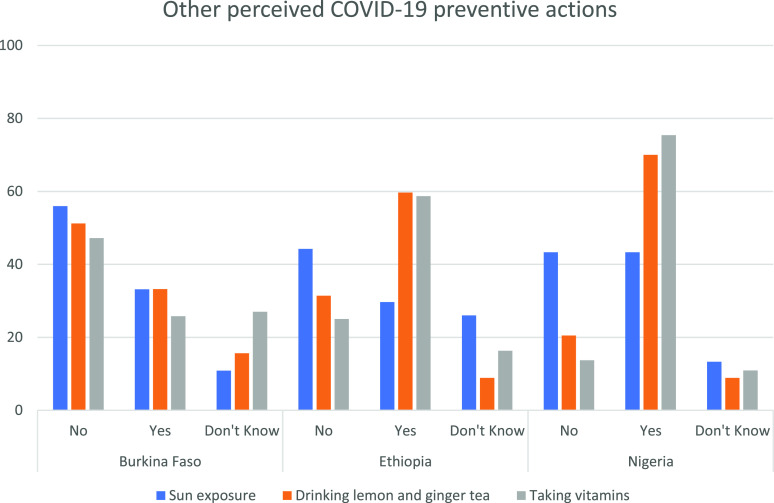
Perceived COVID-19 preventive mechanisms across survey sites in Burkina Faso, Ethiopia, and Nigeria, 2020. This figure appears in color at www.ajtmh.org.

Almost all participants identified the basic preventive methods. However, 31.6% believed that drinking alcohol could prevent COVID-19, especially in Nouna (46%) and Nigeria (38% for both Ibadan and Lagos) (Table 2). The median score for preventive action was higher for Ethiopia (Q1: 6; Q3: 7) (Table 3). Many participants answered that taking vitamins was preventive against COVID-19 (ranging from 75% in Nigeria to 25.8% in Burkina Faso), as was drinking lemon and ginger tea (70% in Nigeria and 30% in Burkina Faso) (Figure 1). Female participants and those in older age groups were more likely to report believing in misinformation about COVID-19, including preventive actions of sun exposure, alcohol drinking, and vitamin supplementation. Educational status (having some primary school, some secondary school, and tertiary education compared with no education), urban residence, and believing the pandemic is real were positively associated with good knowledge of COVID-19 preventive actions (Table 4).

#### Information sources for COVID-19.

**Figure 2.**
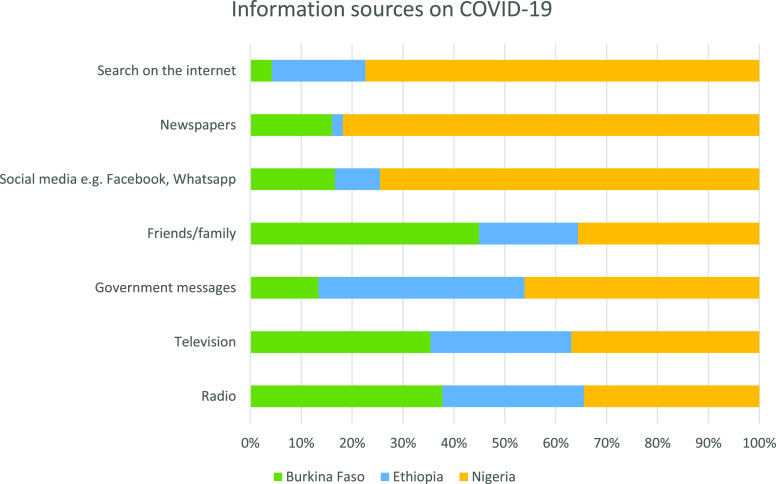
Sources of information related to COVID-19 across the survey sites in Burkina Faso, Ethiopia, and Nigeria, 2020. This figure appears in color at www.ajtmh.org.

Radio and television were the primary sources of information for adults across the three countries. Compared with Ethiopia, Burkina Faso and Nigeria reported higher utilization of these information sources (> 80%) (Table 2 and Figure 2).

### Perception and self-reported prevalence of preventive measures practiced by adults.

Among all participants, 94.6% believed that the COVID-19 pandemic was real. Among those who believed the pandemic is real, only 13.6% knew people who had been sick from COVID-19 (Table 5).

**Table 5:** COVID-19 perception and self-reported preventive measures practiced by adults in Burkina Faso, Ethiopia, and Nigeria, 2020

		Burkina Faso		Ethiopia		Nigeria	
	Total	Nouna	Ouagadougou	Addis Ababa	Kersa	Ibadan	Lagos
Perception, *N* (%)							
Believe COVID-19 pandemic is real							
No	96 (5.4)	17 (5.7)	2 (0.7)	25 (8.7)	13 (4.4)	21 (6.9)	18 (5.8)
Yes	1,700 (94.6)	280 (94.3)	298 (99.3)	263 (91.3)	2,814(95.6)	283 (93.1)	292 (94.2)
Know anyone who has been sick from COVID-19							
No	1,554 (86.5)	264 (88.9)	282 (94.0)	192 (66.7)	285 (96.0)	277 (91.1)	254 (81.9)
Yes	230 (13.6)	33 (11.1)	18 (6.0)	96 (33.3)	12 (4.0)	27 (8.8)	56 (10.1)
Preventive practices, *N* (%)							
Regularly washing hands with soap and water	1,712 (95.3)	289 (97.3)	287 (95.6)	284 (98.6)	255 (85.9)	295 (97.0)	302 (97.1)
Disinfecting surfaces	811 (45.1)	55 (18.5)	74 (24.7)	222 (77.1)	23 (7.7)	234 (77.0)	203 (65.3)
Keeping distance from sick people	1,114 (62.0)	203 (68.4)	151 (50.3)	172 (59.7)	102 (34.3)	253 (83.2)	133 (74.9)
Keeping physical distance from everyone who is not family member	1,213 (67.5)	125 (42.1)	170 (56.7)	211 (73.3)	230 (77.4)	230 (75.7)	247 (79.4)
Stopped going to social gatherings, churches, or mosques	883 (489.1)	128 (43.1)	58 (19.3)	165 (57.3)	159 (54.5)	181 (59.5)	192 (61.7)
Wearing face mask	1,496 (83.3)	207 (69.7)	284 (94.7)	242 (84.0)	236 (79.5)	243 (79.9)	284 (91.3)
Stocking up on food, home supplies and medicine	330 (18.4)	6 (2.0)	12 (4.0)	91 (31.6)	36 (12.2)	78 (25.7)	107 (34.4)
Changing/canceling travel plans	535 (29.7)	85 (28.6)	14 (4.7)	121 (42.0)	13 (4.4)	139 (45.7)	163 (52.4)
Access to clean water and soap, *N* (%)							
For preparing food	1,568 (87.3)	256 (86.2)	239 (79.7)	277 (96.2)	197 (66.3)	290 (95.7)	309 (99.4)
For handwashing	1,742 (97.1)	290 (97.6)	292 (97.3)	280 (97.2)	277 (93.3)	299 (99.0)	304 (98.0)
Have water for handwashing	1,771 (98.7)	290 (97.6)	295 (98.3)	283 (98.3)	296 (99.7)	299 (99.0)	308 (99.0)

Participants reported practicing most of the COVID-19 preventive measures listed in the questionnaire. Regularly washing hands with soap and water and wearing a face mask were the most highly practiced measures by the respondents. Keeping a distance from sick people (62%), avoiding social gatherings (49%), and disinfecting contaminated surfaces (45%) were the least implemented. More than half of the participants in Ethiopia and Nigeria mentioned not attending social gatherings, churches, or mosques, whereas in Ouagadougou, the majority reported still attending social events (Table 5).

#### Alcohol drinking habits and sleep pattern during the COVID-19 pandemic.

**Figure 3.**
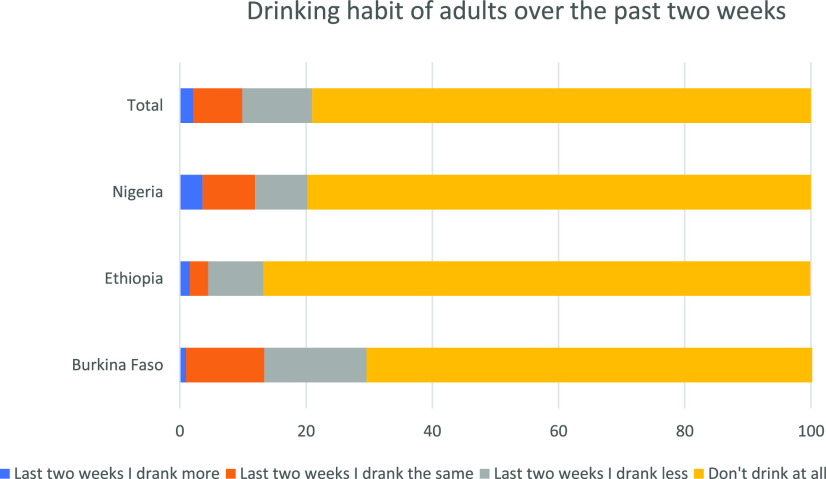
Drinking habit of respondents over the past 2 weeks before the survey in Burkina Faso, Ethiopia, and Nigeria, 2020. This figure appears in color at www.ajtmh.org.

**Figure 4.**
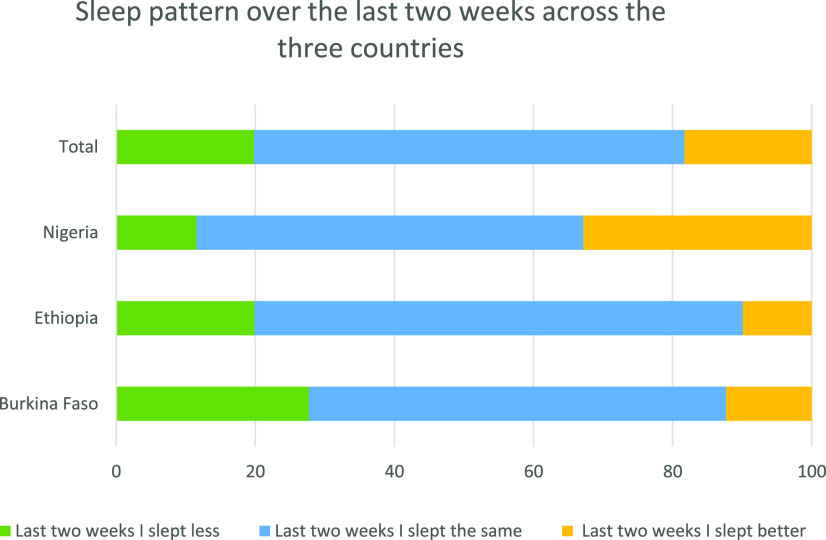
Sleep pattern of adults over the last 2 weeks before the survey in Burkina Faso, Ethiopia, and Nigeria, 2020. This figure appears in color at www.ajtmh.org.

Of the respondents, 70.6% reported that they did not drink alcohol at all (Figure 3). Among those who did report drinking, 2.1% reported drinking more alcohol in the past 2 weeks; among them, 1.8% answered that drinking alcohol is preventive of COVID-19 in the COVID-19 prevention knowledge assessment. Most participants reported that their sleep pattern in the past 2 weeks was similar with their previous experience. More participants in Burkina Faso reported sleeping less than usual in the past 2 weeks than in Nigeria and Ethiopia (Figure 4).

### Mental health status.

The majority of the participants reported not having feelings of nervousness, being anxious, or on edge. Having nervous or anxious feelings for more than half of the days was reported by 6.4% (ranging from 1.7% in Kersa and Lagos to 19.2% in Nouna). Overall, only 4.2% of participants reported feelings of uncontrollable worry nearly every day, ranging from 1.4% in Lagos to 9.1% in Kersa. Similar results were obtained for depression; only 2.5% of participants reported feeling down, depressed, or hopeless nearly every day, and 1.8% mentioned having little interest in doing things nearly every day. For cases of depression, the lowest numbers were reported in Ouagadougou, and the highest were in Addis Ababa. The percentages of participants reporting depression symptoms were slightly higher than for anxiety symptoms (Table 6).

**Table 6:** Impacts of COVID-19 on the mental health of adults in Burkina Faso, Ethiopia, and Nigeria, 2020

		Burkina Faso		Ethiopia		Nigeria	
Impacts of COVID-19	Total	Nouna	Ouagadougou	Addis Ababa	Kersa	Ibadan	Lagos
Feeling nervous, anxious, or on edge, *N* (%)							
Not at all	1,209 (68.8)	220 (74.1)	177 (59.0)	224 (77.8)	185 (62.3)	204 (72.6)	199 (67.5)
Several days	392 (22.3)	17 (5.7)	106 (53.3)	25 (8.7)	91 (30.6)	65 (23.1)	88 (29.8)
More than half the days	113 (6.4)	57 (19.2)	14 (4.7)	26 (9.0)	5 (1.7)	6 (2.1)	5 (1.7)
Nearly everyday	44 (2.5)	3 (1.0)	3 (1.0)	13 (4.5)	16 (5.4)	6 (2.1)	3 (1.0)
Not being able to stop or control worrying, *N* (%)							
Not at all	1,181 (67.2)	222 (74.8)	156 (52.0)	250 (86.8)	156 (52.5)	200 (71.7)	197 (66.6)
Several days	377 (21.5)	17 (5.7)	84 (28.0)	15 (5.2)	110 (37.0)	68 (24.4)	83 (28.0)
More than half the days	125 (7.1)	51 (17.2)	35 (11.7)	18 (6.3)	4 (1.4)	5 (1.8)	12 (4.1)
Nearly everyday	74 (4.2)	7 (2.4)	25 (8.3)	5 (1.7)	27 (9.1)	6 (2.2)	4 (1.4)
Feeling down, depressed, or hopeless, *N* (%)							
Not at all	1,274 (72.5)	209 (70.6)	160 (53.3)	231 (80.2)	257 (86.5)	213 (76.1)	204 (68.9)
Several days	326 (18.6)	20 (6.8)	105 (35.0)	25 (8.7)	34 (11.5)	61 (21.8)	81 (27.4)
More than half the days	113 (6.4)	64 (21.6)	21 (7.0)	17 (5.9)	2 (0.7)	1 (0.4)	8 (2.7)
Nearly everyday	44 (2.5)	3 (1.0)	14 (4.7)	15 (5.2)	4 (1.4)	5 (1.8)	3 (1.0)
Little interest or pleasure in doing things, *N* (%)							
Not at all	1,376 (78.5)	241 (81.1)	226 (75.3)	219 (76.0)	271 (91.3)	215 (77.9)	204 (69.4)
Several days	250 (14.3)	9 (3.0)	62 (20.7)	26 (9.0)	20 (6.7)	55 (19.9)	78 (26.4)
More than half the days	94 (5.4)	45 (15.2)	9 (3.0)	27 (9.4)	2 (0.7)	2 (0.7)	9 (3.1)
Nearly everyday	32 (1.8)	2 (0.7)	3 (1.0)	16 (5.6)	4 (1.4)	4 (1.4)	3 (1.0)
Anxiety, depression, and psychological distress scores (0–6), *N* (%)							
Anxiety (0–6), %							
None (< 3)	1,597 (91.1)	244 (82.4)	271 (90.3)	251 (87.2)	275 (92.6)	270 (97.1)	286 (96.9)
Higher levels of anxiety (≥ 3)	157 (8.9)	52 (17.6)	29 (9.7)	37 (12.8)	22 (7.4)	8 (2.9)	9 (3.1)
Depression (0–6), %							
None (< 3)	1,584 (90.6)	251 (84.5)	263 (87.7)	255 (88.5)	266 (89.6)	266 (97.4)	283 (96.3)
Higher levels of depression (≥ 3)	165 (9.4)	46 (15.7)	37 (12.3)	33 (11.5)	31 (10.4)	7 (2.6)	11 (3.7)
Total psychological distress (0–12), %							
None (0–2)	1,263 (72.3)	224 (75.7)	177 (59.0)	219 (76.0)	239 (80.5)	207 (76.1)	197 (67.2)
Mild (3–5)	360 (20.6)	20 (6.8)	107 (35.7)	47 (16.3)	40 (13.5)	58 (21.3)	88 (30.0)
Moderate (6–8)	104 (5.9)	48 (16.2)	14 (4.7)	17 (5.9)	15 (5.1)	3 (1.1)	7 (2.4)
Severe (9–12)	19 (1.1)	4 (1.4)	2 (0.7)	5 (1.7)	3 (1.0)	3 (1.1)	1 (0.3)

**Figure 5.**
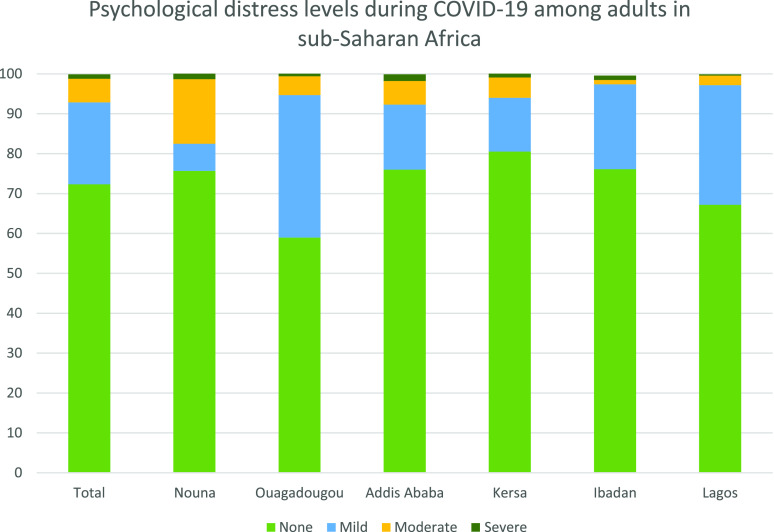
Psychological distress among adults in Burkina Faso, Ethiopia, and Nigeria, 2020. This figure appears in color at www.ajtmh.org.

Most participants (90%) were classified as having no anxiety and depression. Mild, moderate, and severe psychological distress was reported by 20.6%, 5.9%, and 1.1% of the participants, respectively. Ouagadougou had the largest proportion of mild cases (35.7%). The proportion of severe cases was lowest in Lagos (0.3%) and highest in Addis Ababa (1.7%) (Table 6 and Figure 5).

## DISCUSSION

The study assessed knowledge and practices related to COVID-19 among adults and described the levels of depression, anxiety, and psychological distress in rural and urban settings in three SSA countries. We found the levels of knowledge about transmission and prevention to be generally high compared with knowledge about the symptoms of COVID-19. Despite higher levels of knowledge, the proportion of respondents implementing recommended preventive actions was low. Television and radio were the media sources most frequently used to obtain COVID-19 related information. The study also showed higher levels of knowledge of COVID-19 symptoms among male particpants, persons with better educational status, urban residents, and those who believed the pandemic is real. Knowledge of transmission mechanisms was higher among male participants, older age groups, those with higher educational status, urban residence, and those who believed the pandemic is real. Having more education, living in an urban setting, and believing in the pandemic were factors associated with better knowledge of preventive actions. The higher knowledge among educated male participants in urban areas could be mainly due to their access to multiple information sources and a higher demand for COVID-19 information among these groups. Occupation or income were not measured in this survey; however, these could also be related to higher knowledge.

In this study, a large proportion of the general population had a high level of knowledge regarding COVID-19. This result is similar to several other studies conducted in other countries, including those in the sub-Saharan African region.^37,38,43–46^ Our findings suggest higher knowledge than a study from Ethiopia and Nigeria that showed only one-third of adults had good knowledge.^47,48^ This variation could be due to differences in the number and type of questions and the scoring mechanism used to assess knowledge. A study in China used 20 questions with a score of ≥ 16 (80%) to indicate a high level of knowledge,^43^ and a study in Saudi Arabia used 22 questions with a score of ≥ 17.96 (80%).^44^ A study in Cameroon used seven questions with a score of ≥ 4 (57%),^45^ and a score of > 70% was considered in Nigerian study.^38^ A study in Ethiopia used 42 questions, with a cutoff of 80% for classifying a high level of knowledge.^48^ Accordingly, studies that used a smaller number of questions for assessing knowledge along with a lower cutoff value produced exaggerated knowledge proportions. In addition, the differences in results between studies could be due to the methods of data collection; some studies used online platforms, whereas others collected data by distributing questionnaires. Geographic coverage (urban or rural) and the status of the COVID-19 pandemic locally at the time of conducting the studies could also be considered a source of variation.

Previous studies have suggested that an increased level of knowledge is associated with a higher level of protective behaviors that reduce the risk of adverse health conditions.^46,49^ Because having accurate knowledge is key in the prevention and control of COVID-19, risk communication strategies should consider correcting misinformation associated with transmission and prevention modes. The high knowledge scores we observed on the three knowledge domains in this study could be the result of the different public health interventions that were implemented to create public awareness in the sub-Saharan Africa region. We observed that knowledge about COVID-19 symptoms and transmission mechanisms was not as high as knowledge about the prevention measures, which could be because many interventions in place in SSA may focus on disseminating information about prevention measures. Participants reported mosquito bites as one mechanism for COVID-19 transmission. Sun exposure, drinking alcohol and lemon/ginger tea, and taking vitamin supplements were also reported for the prevention of COVID-19. This is consistent with other studies conducted elsewhere, in which participants reported taking herbal tea or medicine is preventive against COVID-19.^50–52^ Accordingly, because there is no clear evidence on the effect of such supplements and herbal treatments, education to correct these myths is needed.

Health information provided to the general population is a fundamental component of prevention and control strategies during pandemics. During past infectious disease outbreaks such as SARS and H1N1, mainstream media were significant sources of information that helped create public awareness.^53,54^ This survey also demonstrated that mainstream media (radio and television) were the main reported sources of information about the pandemic.^38,44–47,55^

Our study showed that, even though the majority of adults identified the recommended preventive measures in the knowledge assessment, the practice of these measures was poor, especially for keeping distance from sick people, avoiding social gatherings, and disinfecting contaminated surfaces. The practice of disinfecting surfaces is much lower in rural sites than in urban sies, which could be due to unaffordability, unavailability of disinfectants, or less attention given to the effect of disinfectants in preventing the transmission of the virus. Although many participants reported not avoiding social gatherings across all three countries, this proportion was relatively higher in Burkina Faso than in Ethiopia or Nigeria. Government regulations restricting social gatherings to 50 or fewer people were in place during the time of data collection in the three countries.^56,57^ This result is similar to other studies done in Nigeria, where 50% of the respondents insisted on attending congregational prayers despite social distancing restrictions.^47^ These findings indicate that further education dissemination and risk communication strategies targeting specific groups are needed to help engage adults in precautionary behaviors. Other studies have found that COVID-19 knowledge had a significant influence on precautionary behavior.^39,49^

In the current study, the majority of study participants mentioned unaltered sleep patterns, which might be because the worst effects of the pandemic are not seen yet in the region because the burden is still lower than other countries in Europe and the Americas.^58^ However, 20% reported sleeping less in the past 2 weeks, with a relatively higher percentage reporting sleeping less in Ouagadougou.

Economic crisis, the threat of unemployment, fear of losing family members, and weak health care systems may lead to immense psychological stress and anxiety in the region during COVID-19.^13,59^ Additionally, disruption of health-related behaviors is also strongly linked with stress, depression, and anxiety.^60^ This study revealed a low level of anxiety and depression among adults in the three countries in SSA, unlike other studies that showed higher percentages of both anxiety and depression.^61–64^ A study conducted in Ibadan revealed higher levels of anxiety related to the pandemic and also suggested that sleeplessness is positively correlated with depression and anxiety.^15^ However, in the current study, few adults reported sleep disturbance, and this could be one reason for the lower levels of depression and anxiety in the current study.

Even though we found low reported percentages of psychological distress in this study, different strategies and interventions are required to address the impact of the pandemic on mental health. To improve accessibility and availability of mental health services, community health workers can be trained to provide mental health education, screening, and counseling services at the community level.^13^

This study has several key strengths. The study included diverse urban and rural sites in three different countries and used a uniform tool and approach across settings to increase comparability across sites. The use of computer-assisted telephone interviewing allowed us to remotely generate high-quality data. Computer-assisted telephone interviewing surveys generate comparable data to those conducted using face-to-face surveys and have the lowest attrition rates compared with other phone survey methods.^65^ This study has several limitations. First, because a mobile survey platform was used for data collection, only households that own mobile phones were included in the survey. However, household cell phone penetration rates in many settings in SSA are high.^66^ Second, all data collected in this survey were self-reported, which limits the conclusions we can make about the actual behavior and practices of the participants. Third, the study populations at each site were not selected to be representative of the larger regional and populations in each country, which limits the generalizability of our conclusions. Nevertheless, the evidence generated in this study is valuable because it provides a clearer picture regarding knowledge, practice, and mental health of the population groups surveyed in Ethiopia, Burkina Faso, and Nigeria.

In conclusion, the majority of the adults surveyed had a high level of knowledge of COVID-19. However, self-reported implementation of preventive mechanisms was lower, especially for avoiding social gatherings, which is among the most important prevention measures. This study also identified common misconceptions related to COVID-19 transmission mechanisms. Public health officials need to formulate or intensify risk communication strategies on COVID-19 using the available communication channels by considering the target audience.

The pandemic is still unfolding, and several waves are expected before the introduction of an effective vaccine at a large scale in SSA. Therefore, there is a strong need to continually generate evidence to better understand the pandemic and related health behaviors in the African population.
